# Handgrip strength during admission for COPD exacerbation: impact on further exacerbation risk

**DOI:** 10.1186/s12890-021-01610-7

**Published:** 2021-07-21

**Authors:** Chi-Tai Lee, Ping-Huai Wang

**Affiliations:** 1 Division of Pulmonology, Department of Internal Medicine, Far Eastern Memorial Hospital, New Taipei City, 220 Taiwan, ROC; 2 Department of Nursing, Oriental Institute of Technology, New Taipei City, Taiwan, ROC

**Keywords:** Handgrip strength, Chronic obstructive pulmonary disease, Exacerbation

## Abstract

**Background:**

Low handgrip strength (HGS) is independently associated with a higher exacerbation risk in stable chronic obstructive pulmonary disease (COPD); however, the relationship between HGS while being admitted for COPD exacerbation and further exacerbation risk after discharge remains unclear.

**Methods:**

We enrolled patients admitted for COPD exacerbation between January 2018 and June 2019. HGS tests were done within 3 days after admission. The primary endpoint was exacerbations within 12 months after the index admission, which needed emergency room visits or hospital admission. We analyzed the relationships among demographics, HGS, pulmonary function parameters, and acute exacerbation events.

**Results:**

Among 43 enrolled patients, 31 (72.1%) participants (HGSw) had HGS weakness (22.1 ± 4.1 kg). The other 12 (27.9%) participants (non-HGSw) had the strength of handgrips 33.7 ± 3.1 kg. HGSw group showed a significantly higher rate of emergency room visits within 6, 9, and 12 months after the index admission than non-HGSw group (0.81 ± 1.30 vs. 0.08 ± 0.29, p = 0.045; 1.26 ± 1.59 vs. 0.17 ± 0.38, *P* = 0.019; 1.48 ± 1.86 vs. 0.25 ± 0.62, *P* = 0.027, respectively). There was a trend to have higher admission rate within 9 and 12 months in HGSw group, which did not achieve statistical significance (0.77 ± 1.38 vs. 0.08 ± 0.29, *P* = 0.064; 0.94 ± 1.56 vs. 0.08 ± 0.29, *P* = 0.062, respectively).

**Conclusions:**

HGS weakness measured upon admission for COPD exacerbation was associated with a higher risk of exacerbation in the next year.

*Trial registration* ClinicalTrials.gov Identifier: NCT04885933.

**Supplementary Information:**

The online version contains supplementary material available at 10.1186/s12890-021-01610-7.

## Background

Chronic obstructive pulmonary disease (COPD) is characterized by persistent respiratory symptoms and airflow limitation, which is caused by the complex interactions among exposure to noxious particles or gases and various host factors, including genetic inheritance, airway hyperresponsiveness, or inadequate lung development [1, 2]. COPD has caused a progressive health burden worldwide mostly due to increasing exposure to noxious particles and the aging population [3]. In Taiwan, the estimated COPD prevalence in individuals aged > 40 years was approximately 6% in 2013 [4]. Moreover, in 2013, chronic airway diseases were the seventh leading mortality cause in Taiwan, with an annual mortality rate of 33.2 per 100,000 population [5].

COPD not only presents lung function impairment but also extrapulmonary complications resulting from systemic inflammation that extends from chronic airway inflammation, including cardiovascular diseases, osteoporosis, and muscle atrophy [6]. Previous studies have shown that skeletal muscle dysfunction increases the risk of COPD morbidity and mortality. Compared with healthy individuals, patients with COPD show reduced strength of skeletal and respiratory muscles. Hamilton et al. reported that 70% of patients with chronic lung disease present with quadriceps muscle weakness [7]. Moreover, patients with moderate-to-severe COPD present with a 20–30% decrease in the quadriceps femoris muscle strength [8–10]. However, muscle dysfunction at COPD might be heterogeneous. Compared to upper limb, the impairment degree of lower limb muscle strength is more correlated with COPD severity [9]. But the possible mechanisms of upper limb weakness was more complicated than lower limbs. It might be associated with respiratory muscle asynchronization and dynamic hyperinflation, not only muscle wasting or impaired endurance caused by COPD, just like lower limbs [11]. Regarding ventilatory muscle function, patients with COPD present with a 30–40% decrease in the maximal diaphragm strength [7, 8]. The heterogeneity and complex of muscle dysfunction in COPD need a simple tool as a good reflection of general muscle strength [12]

Handgrip strength (HGS), not only directly represents hand muscle strength but also is a good surrogate measurement for overall muscle strength [12–14]. Specifically, HGS of COPD patients is correlated with the strength of other muscles, including the quadriceps and respiratory muscles [8, 15]. Not only the representative of muscle strength, the degree of the HGS impairment is associated with a decline in forced expiratory volume in one-second percentage of predicted value (FEV_1_% of predicted value) and COPD Global Initiative for Chronic Obstructive Lung Disease (GOLD) grading [16, 17]. Moreover, Martinez et al. found that a 1-kg reduction in HGS was associated with an increased risk of exacerbation by 5% in stable COPD [18].

However, HSG measurement in these previous studies mostly was for patients with stable COPD. Respiratory discomfort and physical weakness could negatively affect HGS on admission for acute exacerbation of COPD (AECOPD) [19]. To our knowledge, there has been scarce studies on the relationship between HGS in patients admitted with AECOPD and further exacerbation risk. This prospective study aimed to investigate the relationship between HGS in early stage of admissions for AECOPD and post-discharge exacerbation risk of the next year.

## Methods

### Designs and participants

We enrolled patients aged > 45 years who were admitted for AECOPD. COPD was defined as an obstructive ventilatory defect (FEV_1_/FVC < 0.7) based on pulmonary function tests along with smoking for > 15 pack-years or a history of noxious gas exposure or the clinical impression by attending physicians in cases that spirometry data were missing. Acute exacerbation was defined as acute worsening of respiratory symptoms that results in additional therapy. We excluded patients with heart failure; permanent pacemaker or implantable cardioverter-defibrillator [20]; significant fluid retention, including edema, pleural effusion, or ascites; morbid obesity with BMI > 34 [21]; use of noninvasive positive-pressure ventilators (NIPPV) use upon admission; structural lung defects by chest plain films, including significant tuberculosis sequelae, bronchiectasis, and pneumoconiosis; and re-admission for AECOPD within 1 month. Participants received standard care based on the clinical judgments of attending physicians.

The COPD assessment test (CAT) questionnaire was simultaneously administered. Data regarding exacerbation occurrence with emergency room visits or even admission were obtained at three-month intervals through outpatient clinic follow-up visits, medical record or telephone interviews. The CAT questionnaire was re-administered at the third post-discharge month. The primary end-points were acute exacerbations leading to emergency room visits or readmission within 12 months after admission. This study was approved by the Institutional Review Board of Far Eastern Memorial Hospital (FEMH-10699-E). In addition, participants had to sign informed consents before initiating the study.

### Handgrip strength measurement

HGS measurements were performed within 3 days of admission. HGS measurements were performed by using a dynamometer (North Coast Hydraulic Hand Dynamometer, North Coast Medical Inc., Morgan Hill, CA). The patient was seated with the wrist neutrally positioned and the elbow flexed at 90 degree[22]. For patients who were unable to sit, HGS measurements were obtained while lying in bed at 30° with supported elbows. HGS was measured by the dominant hand of participants three times with the interval of at least one minute. The highest value was used in our analyses. HGS weakness (HGSw) was defined based on the guidelines of the European Working Group on Sarcopenia in Older People (EWGSOP) [23]. The patients were divided into the HGSw and non-HGSw groups, with cut-off HGS values of < 30 kg in men and < 20 kg in women [23].

### Measurement of skeletal muscle mass

Skeletal muscle mass was assessed using a foot-to foot bioelectrical impedance analyzer (BIA) (TBF-410-GS Tanita, Japan). It was measured, following by the manufacture’s instruction.

### Sarcopenia definition

Sarcopenia was defined based on EWGSOP recommendations as follows: skeletal muscle index (skeletal muscle mass/height^2^, SMI) of men was less than 8.87 kg/m^2^ and that of women was < 6.42 kg/m^2^ in conjugated to HGS weakness [23].

### Pulmonary function test

Pulmonary spirometry data, including FEV1, forced vital capacity (FVC), and FEV1/FVC, were collected within 12 months before admission according to electronic medical record. All presented data were post-bronchodilator data.

### Statistical analysis

All statistical analyses were used IBM SPSS statistics Version 19. Categorical and continuous variables were compared using the chi-square and Mann–Whitney U tests, respectively. One-way analysis of variance (ANOVA) was used for between-group comparison of pulmonary obstruction severity, which was graded using the GOLD classification. Cox regression analysis was used for the time to the first emergency room visit or first readmission after discharge. Statistical significance was set as P < 0.05.

## Results

**Fig. 1:**
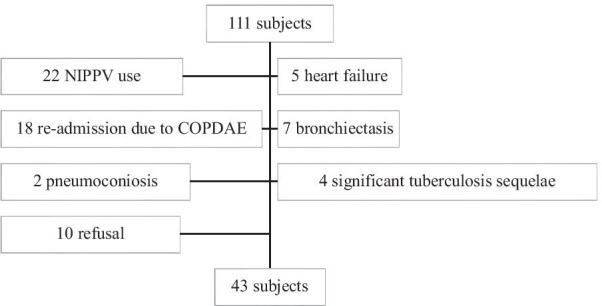
Flowchart of inclusion of study participants

There were 111 participants admitted for AECOPD who were potentially eligible. After screening based on the exclusion criteria and willingness to participate, we finally enrolled 43 participants (Fig. 1). Table 1 presented the demographic characteristics of the participants. Among the 43 participants, 31 (72.1%) participants belonged to HGSw group. The HGS was significantly lower in HGSw than non-HGSw group(22.1 ± 4.1 kg vs. 33.7 ± 3.1 kg, *P* < 0.001). There were no between-group differences in age; sex; and comorbidities, including hypertension, diabetes, and heart diseases (coronary artery disease and valvular heart disease) (Table 1). However, compared with the HGSw group, the non-HGSw group had more active smokers (9.7% vs. 50%, *P* = 0.003). As expected, BMI and SMI were significantly higher in the non-HGSw group than in the HGSw group. But all participants did not reach the criteria of sarcopenia.

**Table 1:** Demographic characteristics of the participants

	Total (n = 43)	HGSw (n = 31)	Non-HGSw (n = 12)
Gender(M/F)	(40/3)	28/3	12/0
Age	72.3 ± 9.2	75.2 ± 7.3	65.1 ± 9.8
Smoking (+/−/ex) n(%)	9/3/31 (20.9/7/72.1)	3/3/25 (9.7/9.7/80.6)	6/0/6 (50/0/50)*
Hypertension n(%)	18 (41.9)	14 (45.2)	4 (33.3)
CVA n(%)	1 (2.3)	1 (3.2)	0 (0)
DM n(%)	8 (18.6)	5 (16.1)	3 (25.0)
Heart disease n(%)	10 (23.3)	7 (22.6)	3 (25.0)
CAD	8 (18.6)	5 (16.1)	3 (25.0)
VHD	2 (4.6)	2 (6.4)	0 (0)
CKD n(%)	1 (2.3)	1 (3.2)	0 (0)
Cancer n(%)	2 (4.7)	2 (6.5)	0 (0)
Exacerbation in previous year	0.67 ± 1.36	0.74 ± 1.53	0.50 ± 0.80
BMI (kg/m^2^)	23.3 ± 4.3	22.3 ± 4.2	25.8 ± 3.6*
SMI (kg/m^2^)	17.2 ± 2.0	16.5 ± 1.8	18.8 ± 1.3*
HGS (kg)	25.3 ± 6.5	22.1 ± 4.1	33.7 ± 3.1*
Hospitalization duration (day)	8.2 ± 5.2	8.84 ± 5.8	6.5 ± 2.4

BMI, body mass index; CKD, chronic kidney disease; CVA, cerebrovascular accident; DM, diabetes mellitus; ex, ex-smoker; F, female; HGS, handgrip strength; HGSw, handgrip strength weakness; M, male; SMI, smooth muscle index

^*^ Significant difference within groups HGSw vs. non-HGSw (*P* < 0.05)

Nearly total participants (41/43, 95.3%) ever had lung function tests to confirm the diagnosis of COPD. Only two of them, belonged to HGSw group, had no adequate lung function data because they only achieved submaximal effort. 30 participants had lung function data within one year of recruitment. 21(67.7%) and 9(75%) participants were in the HGSw and non-HGSw groups, respectively (Additional file 1: Table S1). FEV_1_, FEV_1_ predicted %, forced vital volume (FVC), and FVC predicted % were significantly lower in the HGSw group than in the non-HGSw group. There was significant difference in the severity of GOLD grade between HGSw and non-HGSw groups (*P* = 0.002) (Additional file 1: Table S1).

**Fig. 2:**
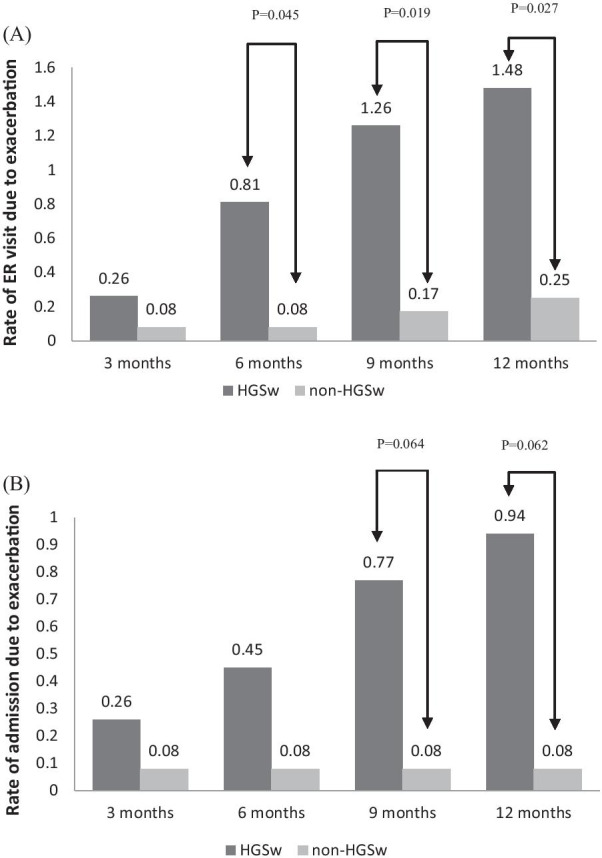
Rate of exacerbations requiring emergency room visit and admission within three, six, nine, and twelve months of the index admission. Abbreviation: ER: emergency room; HGSw: handgrip strength weakness. (**A**) The rates of emergency room visits within 6, 9, and 12 months after index admission were significantly higher in the HGSw group than in the non-HGSw group. (**B**) It did not achieve significant differences in the readmission rates within 3, 6, 9 or 12 months between HGSw and non-HGSw groups. There was a trend to have higher admission rate within 9 and 12 months in HGSw group, even though there was no statistical significance

There was no significant between-group difference in the CAT score at admission (CAT_ad_) and 3 months after index admission (CAT_3m_) (10.2 ± 4.3 vs 13.8 ± 5.9, *P* = 0.272) (Table 2). Significantly clinical difference between CAT_ad_ and CAT_3m_ was defined as the difference with equal or more than 2. It was not significantly different between HGSw and non-HGSw groups (35.5% vs. 41.7%, *P* = 0.757). Compared with non-HGSw group, the HGSw group showed significantly higher rates of emergency room visits within 6 (0.81 ± 1.30 vs. 0.08 ± 0.29, *P* = 0.045), 9 (1.26 ± 1.59 vs. 0.17 ± 0.38, *P* = 0.019), and 12 months after index admission (1.48 ± 1.86 vs. 0.25 ± 0.62, *P* = 0.027). However, it did not achieve significant differences in the readmission rates within 3, 6, 9 or 12 months between HGSw and non-HGSw groups (Table 2, Fig. 2). There was a trend to have higher admission rate within 9 and 12 months in HGSw group, even though there was no statistical significance (0.77 ± 1.38 vs. 0.08 ± 0.29, *P* = 0.064; 0.94 ± 1.56 vs. 0.08 ± 0.29, *P* = 0.062, respectively). The time to the first emergency room visit or first readmission after discharge was not significantly early in HGSw group, compared to non-HGSw group (Additional file 2: Fig. S1).

**Table 2:** CAT score on admission and after three months, as well as the rate of emergency room visits and admissions within three, six, and twelve months after index admission

	Total (n = 43)	HGSw (n = 31)	non-HGSw (n = 12)	*P*
CAT_ad_	16.6 ± 4.3	16.7 ± 4.3	16.5 ± 4.7	0.817
CAT_3m_	12.9 ± 5.6	13.8 ± 5.9	10.2 ± 4.3	0.272
CAT_ad-3 m_≧2 n (%)	16 (37.2)	11 (35.5)	5 (41.7)	0.737
ER_3m_	0.20 ± 0.46	0.26 ± 0.51	0.08 ± 0.29	0.279
Admission_3m_	0.20 ± 0.51	0.26 ± 0.58	0.08 ± 0.29	0.364
ER_6m_	0.60 ± 1.15	0.81 ± 1.30	0.08 ± 0.29	0.045
Admission_6m_	0.34 ± 0.81	0.45 ± 0.93	0.08 ± 0.29	0.188
ER_9m_	0.95 ± 1.44	1.26 ± 1.59	0.17 ± 0.38	0.019
Admission_9m_	0.58 ± 1.21	0.77 ± 1.38	0.08 ± 0.29	0.064
ER_12m_	1.13 ± 1.69	1.48 ± 1.86	0.25 ± 0.62	0.027
Admission_12m_	0.69 ± 1.38	0.94 ± 1.56	0.08 ± 0.29	0.062

Admission_3m_: admission during three months after index admission; Admission_6m_: admission during 6 months after index admission; Admission_9m_: admission during 6 months after index admission; Admission_12m_: admission during 12 months after index admission; CAT: chronic obstructive pulmonary disease assessment test; CAT_ad_: CAT on admission; CAT_3m_: CAT three months after index admission; CAT_ad-3 m_: the difference of CAT_ad_ minus CAT_3m_; ER_3m_: emergency room visits during three months after index admission; ER6_m_: emergency room visit during 6 months after index admission; ER9_m_: emergency room visit during 6 months after index admission; ER_12m_: emergency room visit during 12 months after index admission; HGS, handgrip strength; HGSw, handgrip strength

## Discussion

Martinez et al. reported HGS was associated with exacerbation risk in cross-sectional and longitudinal analyses [18]. However, the relationship of inpatient HGS measurement with further exacerbation risk was little investigated. Regarding physical weakness and respiratory distress in admission, HGS assessment is rarely recommended at acute stage of AECOPD admission, though there is small-scale studies reported about HGS in intensive care and respiratory failure [24]. We observed an association of HGSw with emergency department visits within 6 to 12 months after index admission, which could be possibly related to higher admission rates in the subsequent 9 to 12 months. This suggests that HGSw could be associated with the risk of further AECOPD requiring medical emergency or admission care.

The study about HGS measurement in ICU suggested HGS data might vary as clinical course changed [24]. There might be time-dependent about HGS measurement. The reason that we chose early stage of admissions due to COPDAE, was that HGS was feasible in hospitalized patients even though in intensive care unit [24]. Even if HGS is measured at pre-discharge stage, it also might be confounding by hospital stay, physical inactivity and other possible complications. And another possible benefit of HGS measurement in early admission is to provide the information of muscle weakness early and might arrange pulmonary rehabilitation in adequate timing of hospital stay.

The exacerbation risk is associated with the exacerbation history within the previous year and COPD GOLD grading [25]. However, we observed no between-group difference in the exacerbation history in the previous year. The airflow limitation degree could confound the relationship between HGSw and further exacerbation. However, lung function tests are not routinely recommended in the acute stage of AECOPD [1]. Lung function might be underestimated in the acute stage of patients showing insufficient effort and cooperation due to physical weakness. A similar concern could be associated with HGS. Nonetheless, even without the data of lung function tests, the present study showed that inpatient HGS measurement might be a predictor of further exacerbation.

Kaymaz et al. [26] reported a significant association of upper limb muscle strength with exercise capacity, dyspnea sensation, and quality of life in patients with severe COPD. Impaired upper limb muscle strength might be indicative of HGS weakness. Consistent with this, HGS is significantly associated with exacerbation rates and the severity of airflow obstruction among patients with stable COPD [16–18]. In addition to upper limb muscle strength, HGS is associated with lower limb strength and the 6-min walk distance [8]. HGS weakness was also reported to associate with increased mortality risk [27]. We observed a correlation of HGS weakness with the risk COPD re-exacerbation rates in need of emergency care from half to one year, and it possibly increased re-admission rate of exacerbation. HGS weakness of inpatients, admitted due to AECOPD, might be a predictor of exacerbation risk in the next year even in the status of lacking lung function data. Upper limb training, which could improve arm function and reduce symptoms in patients with COPD, should be considered for patients with HGSw and difficulties in daily arm activities [28–30]. Therefore, the HGS of inpatients might not only provide additional treatment clues regarding pulmonary rehabilitation but also act as a follow-up parameter.

This study has several limitations. First, the study had a small number of participants. The statistical power was limited. And we excluded patients with apparent fluid overload and severe exacerbation under NIPPV use on admission, which may not reflect the real population. There is a need for future large-scale studies to yield more comprehensive results. Second, the report showed BMI and skeletal muscle index were significantly different between HGSw and non-HGSw groups. However, the measurement of body weight and height at stable gesture might not be feasible on the scenarios of the first admission days due to acute stage of exacerbation. The study designed to investigate the relationship of handgrip strength in acute stage of admissions on further exacerbation subsequently one year. Therefore, we focused on the variable of handgrip strength to analysis in our study. And Maritnez et al. in 2017 about HGS in COPD, used regression models adjusted for multiple variables, which reported that HGS in stable patients was associated with exacerbation risk [18]. Third, the timing and cooperation of measuring HGS might be confounding factors in acute stage of AECOPD admission.

## Conclusions

In conclusion, we observed that HGSw in inpatients with AECOPD was associated with a higher risk of further exacerbations, not only providing the information of limb muscle strength.

## Supplementary Information

**Additional file 1: Table S1.** Post-bronchodilator lung function test and COPD GOLD stage of the participants within one year before recruitment

**Additional file 2: Figure S1.** Cox regression analysis of (A) time to first emergency room visit and (B) time to first readmission after discharge

## Data Availability

The data that support the findings of this study are available from the corresponding author, [P.H.W], upon reasonable request.

## Abbreviations

HGS: Handgrip strength
COPD: Chronic obstructive pulmonary disease
FEV_1_: Forced expiratory volume in one second
BMI: Body mass index
AECOPD: Acute exacerbation of COPD
NIPPV: Noninvasive positive pressure ventilators
CAT: COPD assessment test
EWGSOP: European Working Group on Sarcopenia in Older People
BIA: Bioelectrical impedance analyzer
SMI: Skeletal muscle index
FVC: Forced vital volume
HGSw: HGS weakness
KNHANES: Korean National Health and Nutrition Examination Survey
