# Supplementary material for: Handgrip strength during admission for COPD exacerbation: impact on further exacerbation risk
Source: BMC Pulm Med. 2021 Jul 21;21:245. doi: 10.1186/s12890-021-01610-7 (PMC8296662; doi:10.1186/s12890-021-01610-7)
Supplement: Supplementary file 1 — Additional file 1: Table S1. Post-bronchodilator lung function test and COPD GOLD stage of the participants within one year before recruitment [file 12890_2021_1610_MOESM1_ESM.docx]

Supplementary Table 1: Post-bronchodilator lung function test and COPD GOLD stage of the participants within one year before recruitment

|  | Total  (n = 43) | HGSw  (n = 31) | non-HGSw  (n = 12) | p |
| --- | --- | --- | --- | --- |
| PFT exam | 30(69.7) | 21(67.7) | 9(75.0) |  |
| FVC | 2.0 ± 0.81 | 1.82 ± 0.69 | 2.65 ± 0.81 | 0.017 |
| FVC predicted % | 63.9 ± 18.8 | 56.2 ± 14.1 | 77.0 ± 21.6 | 0.023 |
| FEV_1_ | 1.05 ± 0.56 | 0.82 ± 0.20 | 1.59 ± 0.77 | 0.018 |
| FEV_1_ predicted % | 45.2 ± 20.2 | 36.2 ± 10.4 | 66.3 ± 22.2 | 0.004 |
| FEV_1_/FVC (%) | 52.1 ± 12.7 | 50.0 ± 12.5 | 57.0 ± 12.4 |  |
| GOLD grade |  |  |  | 0.002 |
| I (n) | 2 | 0 | 2 |  |
| II (n) | 9 | 4 | 5 |  |
| III (n) | 13 | 12 | 1 |  |
| IV (n) | 7 | 6 | 1 |  |

**Abbreviation：** FEV_1_: forced expiratory volume in one second; FEV_1_ predicted %: forced expiratory volume in one-second percentage of predicted value; FVC: forced vital volume; FVC predicted %: forced vital capacity percentage of predicted value; GOLD: global initiative of chronic obstructive lung disease; HGS, handgrip strength; HGSw, handgrip strength weakness; PFT: pulmonary function test
