# Supplementary material for: Handgrip strength during admission for COPD exacerbation: impact on further exacerbation risk
Source: BMC Pulm Med. 2021 Jul 21;21:245. doi: 10.1186/s12890-021-01610-7 (PMC8296662; doi:10.1186/s12890-021-01610-7)
Supplement: Supplementary file 2 — Additional file 2: Figure S1. Cox regression analysis of (A) time to first emergency room visit and (B) time to first readmission after discharge [file 12890_2021_1610_MOESM2_ESM.docx]

Supplementary Figure 1: Cox regression analysis of (A) time to first emergency room visit and (B) time to first readmission after discharge

(A)

**
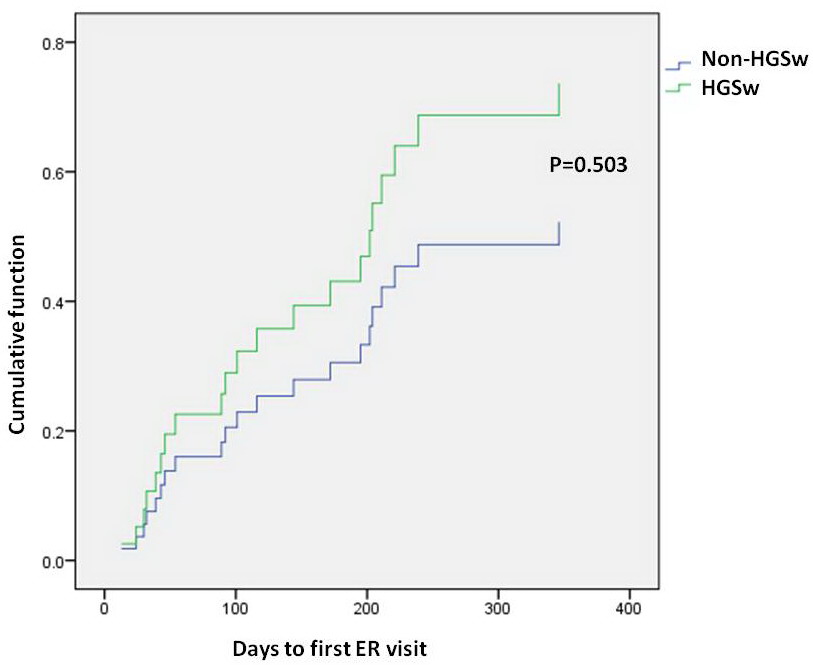
**

**(B)**

**
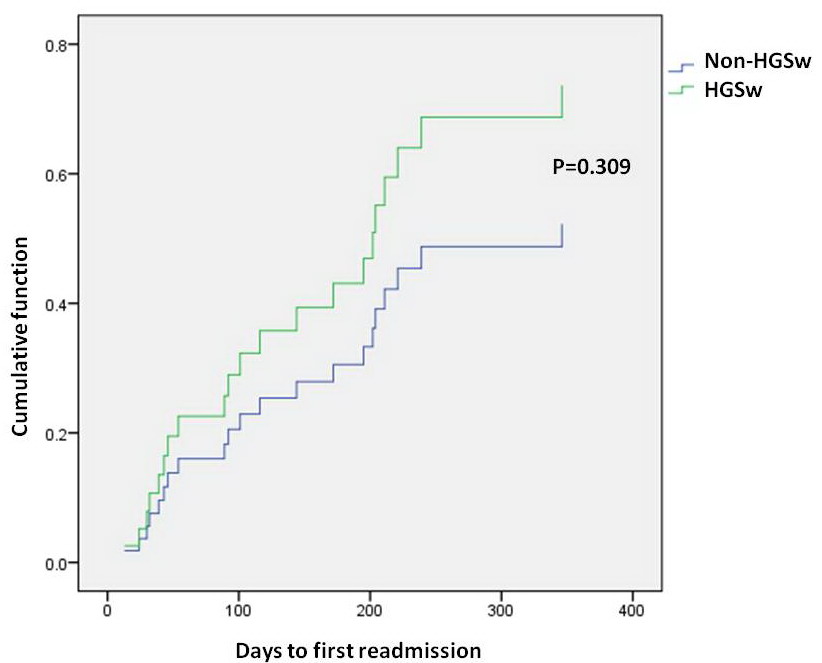
**

**Abbreviation：** HGS, handgrip strength; HGSw, handgrip strength weakness; PFT: pulmonary function test;ER:emergency room
